# Deficient Suppression of Default Mode Regions during Working Memory in Individuals with Early Psychosis and at Clinical High-Risk for Psychosis

**DOI:** 10.3389/fpsyt.2013.00092

**Published:** 2013-09-10

**Authors:** Susanna L. Fryer, Scott W. Woods, Kent A. Kiehl, Vince D. Calhoun, Godfrey D. Pearlson, Brian J. Roach, Judith M. Ford, Vinod H. Srihari, Thomas H. McGlashan, Daniel H. Mathalon

**Affiliations:** ^1^Psychiatry, University of California San Francisco, San Francisco, CA, USA; ^2^Mental Health Service, San Francisco VA Medical Center, San Francisco, CA, USA; ^3^Psychiatry, Yale University, New Haven, CT, USA; ^4^Psychology, University of New Mexico, Albuquerque, NM, USA; ^5^Translational Neuroscience, The Mind Research Network, Albuquerque, NM, USA; ^6^Electrical and Computer Engineering, University of New Mexico, Albuquerque, NM, USA; ^7^Olin Neuropsychiatry Research Center, Institute of Living, Hartford, CT, USA; ^8^Neurobiology, Yale University, New Haven, CT, USA

**Keywords:** schizophrenia prodrome, ultra-high-risk youth, dorsolateral prefrontal cortex, fMRI task-induced deactivation, adolescent mental health

## Abstract

**Background:** The default mode network (DMN) is a set of brain regions typically activated at rest and suppressed during extrinsic cognition. Schizophrenia has been associated with deficient DMN suppression, though the extent to which DMN dysfunction predates psychosis onset is unclear. This study examined DMN suppression during working memory (WM) performance in youth at clinical high-risk (CHR) for psychosis, early schizophrenia (ESZ) patients, and healthy controls (HC). We hypothesized that the DMN would show load-dependent suppression during WM retrieval in HC but not in ESZ, with CHR participants showing an intermediate pattern.

**Methods:** fMRI data were collected from CHR (*n* = 32), ESZ (*n* = 22), and HC (*n* = 54) participants, ages 12–30. DMN regions were defined via seed-based connectivity analysis of resting-state fMRI data from an independent HC sample. Load-dependent deactivations of these DMN regions in response to WM probes were interrogated.

**Results:** Healthy controls showed linear load-dependent increases in DMN deactivation. Significant Group-by-Load interactions were observed in DMN regions including medial prefrontal and lateral posterior parietal cortices. Group-by-Load effects in posterior DMN nodes resulted from less suppression at higher WM loads in ESZ relative to HC, with CHR differing from neither group. In medial prefrontal cortex, suppression of activity at higher WM loads was significantly diminished in both CHR and ESZ groups, relative to HC. In addition, investigation of dorsolateral prefrontal cortex (DLPFC) activations revealed that ESZ activated right DLPFC significantly more than HC, with CHR differing from neither group.

**Conclusion:** While HC showed WM load-dependent modulation of DMN suppression, CHR individuals had deficient higher-load DMN suppression that was similar to, but less pronounced than, the distributed suppression deficits evident in ESZ patients. These results suggest that DMN dysregulation associated with schizophrenia predates psychosis onset.

## Introduction

Deviations from normative patterns of task-induced brain activity have been widely described in schizophrenia, particularly in the context of prefrontally mediated executive functions (1, 2). More recently, research focused on the functioning of cortical circuits during non-task “rest” states indicates that dynamic interactions between networks specialized for extrinsic versus intrinsic processing may be helpful in understanding cognitive impairment in schizophrenia [for review, see Ref. (3, 4)]. The default mode network (DMN) comprises a set of brain regions whose coordinated, synchronous activity is greater during rest than during extrinsic task performance (5). The DMN was characterized incidentally when researchers who were studying task-related activations observed a set of regions showing consistent decreases in activation (i.e., “deactivation”) across a wide range of cognitively demanding tasks (6–8). The regions that most robustly constitute the DMN are medial prefrontal cortex (mPFC) (Brodmann areas 9/10, anterior cingulate cortex), posterior cingulate/retrosplenial cortex, and bilateral lateral parietal cortices (inferior parietal lobules, including Brodmann area 39) (5, 9). In addition, bilateral hippocampal formation and inferior lateral temporal cortices have been identified as part of the DMN, albeit less consistently, which may reflect accessory subsystems within the network (10). Although the precise function of the DMN remains an area of active investigation, its greater activation during non-task rest states has led researchers to speculate that the network mediates stimulus-independent and self-relevant mental activity, possibly in the service of integrating past experiences and planning for future events (10, 11).

The magnitude of task-induced deactivation within the DMN increases with task difficulty, which may reflect a reallocation of processing resources to prioritize circuitry most relevant to supporting task goals (12, 13). Moreover, individual variation in the extent to which DMN activity is suppressed during extrinsic task engagement relates to behavioral outcomes. Greater task-induced deactivation (i.e., suppression) of DMN regions positively correlates with cognitive performance in healthy individuals (12, 14), while less task-induced DMN deactivation is associated with greater self-report of “mind-wandering” (15). This suggests that optimal performance on cognitively challenging tasks may rely, at least in part, on efficient suppression of the DMN. Indeed, failure of adequate DMN suppression has been implicated in mechanisms of attentional lapse and interference of spontaneous cognition on effortful information processing (16, 17). Existing data suggest that both functional integrity within the DMN as well as the reciprocal (i.e., anti-correlated) relationship between the DMN and task-positive networks (18), are potentially important factors in understanding variability in cognitive performance. For example, reductions of both DMN suppression and task-positive network activation predicted performance errors on a speeded attention task; that is, subsequent performance errors were presaged by diminished DMN deactivation in addition to decreased task-relevant regional activation (19). These phenomena may bear particular relevance to schizophrenia (4, 20), a disease with hallmark deficits in attentional, executive, and self-monitoring abilities, underscoring the behavioral relevance of studying task-induced deactivation within the DMN in schizophrenia.

### Default mode network abnormalities in schizophrenia

Previous research has demonstrated functional abnormalities within the DMN in schizophrenia. A number of studies have reported reduced task-induced deactivation (i.e., deficient suppression) of the DMN in schizophrenia, relative to controls (4, 21, 23–25, 27). To date, DMN-focused research has largely studied chronic schizophrenia patients, although decreased task-induced DMN deactivation has recently been reported in a sample of remitted first-episode patients (26). Importantly, findings of deficient DMN suppression in schizophrenia, particularly in mPFC, have withstood control for task performance (24, 25, 27), suggesting that between-group differences in task-induced DMN deactivation are not simply the result of performance differences between schizophrenia patients and healthy comparison subjects. Though methodologically distinct from studies of task-induced deactivation, several analyses of functional connectivity (i.e., temporal correlations of fMRI time-series data between brain regions) have shown resting-state hyperconnectivity between DMN regions in schizophrenia patients relative to healthy controls (HC) (25, 28, 29), although decreased connectivity between posterior cingulate and other DMN regions (30) as well as hyperconnectivity of posterior DMN nodes in combination with hypoconnectivity of frontal nodes (22) have also been reported. In addition, studies examining connectivity among functional networks have reported that the DMN is one of several resting-state networks in schizophrenia that shows altered cross-network functional coupling (29, 31, 32). Thus, while there is strong evidence for abnormal DMN functional connectivity in schizophrenia, consistent with theories that propose dysconnectivity as a central pathophysiological mechanism for the disorder (33–35), there is not complete consensus about the direction of connectivity abnormalities. Association of symptom severity ratings with DMN function has revealed relationships between psychotic symptoms and alterations of DMN connectivity (25, 30) and activity (21). In addition, reduced medial prefrontal task-related suppression, and increased medial prefrontal cortical (25) and inferior temporal connectivity with the DMN (28) have been observed in unaffected first-degree relatives of patients with schizophrenia. Similarly, a statistical trend toward reduced DMN connectivity as a function of cognitive load has been reported in schizophrenia patients and their siblings, relative to HC and their siblings (36). Findings pointing to DMN dysfunction in relatives of patients with schizophrenia suggest that at least some aspects of altered DMN function may be an endophenotypic marker, rather than a direct correlate of frank illness.

### Default mode network activity and brain development

Neurodevelopmental studies suggest maturation and refinement of the functional relationship among DMN nodes from childhood into adulthood, indicating that the DMN may become more cohesive and specialized with age. Similar DMN anatomy, but decreased DMN functional connectivity, have been observed in typically developing children relative to adults (37). Along with findings of increased DMN correlation strength with age (38), these results suggest that the DMN develops and consolidates over the course of neurodevelopment. Moreover, brain maturation from early adolescence to adulthood appears to involve not only strengthening of within-network connectivity, but also diminution of cross-network connectivity across multiple networks including the DMN (39). These changes in within- and across-network functional connectivity associated with adolescence may reflect underlying neuromaturational processes such as synaptic pruning (40, 41) and axonal myelination (42, 43) that are ongoing during the adolescent period and are thought to underlie the final stages of cognitive development. Accordingly, it is important to take normal brain maturation effects on DMN function into account when studying clinical disorders that typically emerge during adolescence and early adulthood.

### Neurodevelopment, pathogenesis of schizophrenia, and default mode network function

The study of the pathogenesis of schizophrenia, including recent interest in the schizophrenia prodrome, has focused on the adolescent period because it is during this developmental window that psychosis typically emerges. Consistent with a neurodevelopmental hypothesis of schizophrenia (44–47), abnormal neuromaturational processes during the adolescent/young adult period have been implicated in the manifestation of schizophrenia. Several candidate mechanisms linking abnormal brain maturation during adolescence/young adulthood to the development of full-blown schizophrenia have been proposed, including excessive pruning of synaptic connections (45, 48) distributed dysconnectivity (33, 34) or, more specifically, dysregulation of NMDA receptor-mediated synaptic plasticity (35). This body of developmentally focused work supports a focus on examining brain functioning in adolescents and young adults who are at clinical risk for psychosis, or who are early in the illness course of schizophrenia.

The advent and validation of criteria for prospective identification of individuals at clinical high-risk (CHR) for schizophrenia and other psychotic disorders (49–53) has permitted the field to make advances in studying functional brain abnormalities in CHR individuals, allowing examination of which abnormalities precede psychosis onset and which arise after the onset of a full-blown psychotic disorder [for review, see Ref. (54)]. However, to date, little is known about whether the altered DMN function observed in patients with schizophrenia is evident in CHR individuals prior to psychosis onset. One previous study of CHR youth conducted a functional connectivity analysis of resting-state data and found hyperconnectivity within the DMN as well as reduced anticorrelations between the DMN and task-positive network regions including the dorsolateral prefrontal (DLPFC) and inferior parietal cortices (55). However, to our knowledge, there has been no prior evaluation focused on task-induced deactivation of the DMN within a sample of individuals at CHR for psychosis.

Accordingly, the present study examined the extent to which the deficient task-induced deactivation of the DMN previously reported in schizophrenia is present in individuals at CHR for psychosis and in schizophrenia patients early in their illness course. Specifically, CHR adolescents and young adults, early schizophrenia (ESZ) patients who were within 5 years of conversion to a schizophrenia diagnosis, and demographically matched HC performed a multi-load working memory (WM) fMRI task based on the paradigms developed by Sternberg (56). In addition to our primary analytic focus on DMN deactivation patterns, a targeted analysis of task-related activations was undertaken based on strong implication of DLPFC involvement in WM performance, and prior demonstration of DLPFC dysfunction in schizophrenia [for example, Ref. (2, 57–61)]. We hypothesized that the HC group would show increasing DMN suppression with load during WM retrieval, consistent with previous literature (12, 24, 25, 62). In contrast, we expected that the normative pattern of DMN suppression with increasing WM load would be significantly diminished in ESZ. Further, we expected that CHR participants would show an intermediate pattern between that of HC and ESZ, consistent with prior findings regarding other aspects of brain functioning in CHR that suggest an intermediate phenotype of brain dysfunction (63).

## Materials and Methods

### Participant recruitment and clinical assessment

Clinical high-risk participants (*n* = 32; 21 male, 11 female) were recruited from Yale University’s PRIME (Prevention through Risk Identification, Management, and Education) clinic, which specializes in identifying and treating individuals experiencing potentially prodromal symptoms and signs of psychosis. CHR patients met the Criteria of Prodromal Syndromes (COPS) based on the Structured Interview for Prodromal Syndromes (SIPS) (50, 64) administered by a trained rater. COPS criteria comprise three non-mutually exclusive CHR sub-syndromes: attenuated psychotic symptoms, brief intermittent psychotic states, and/or genetic risk with deterioration in social/occupational functioning. Detailed descriptions of SIPS symptom severity scales, risk syndrome diagnostic criteria, and psychometric properties are available (50, 64–72). Current clinical criteria and instruments for diagnosing the prodromal syndrome show strong diagnostic validity (72), with conversion to a psychotic disorder occurring in about 35% of CHR patients over a 2- to 3-year follow-up period (73, 74). All CHR participants in our sample met COPS criteria for attenuated psychotic symptoms. Clinical ratings of current symptom severity in CHR patients were obtained by trained raters using the Scale of Prodromal Symptoms (SOPS), which is an embedded scale within the SIPS. CHR participants were all antipsychotic medication-naïve.

Early schizophrenia patients (*n* = 22; 16 male, 6 female) were recruited by referral from clinicians in the greater New Haven community and were required to be within 5 years of initial treatment for psychosis (e.g., hospitalization, diagnosis, or antipsychotic medication treatment). Diagnosis of schizophrenia or schizoaffective disorder was confirmed in ESZ participants via interview by trained raters using the Structured Clinical Interview for DSM-IV (SCID) (75) and current symptom severity was rated using the Positive and Negative Syndrome Scale (PANSS) (76). The majority of ESZ patients (18/22) were being treated with antipsychotic medication at the time of study participation.

Healthy controls participants (*n* = 54; 34 male, 20 female) were recruited from the local community via newspaper, flyer, and brochure advertisements, and did not meet criteria for any current or lifetime Axis I diagnoses based on a structured interview using the SCID (for HC participants >16 years of age) or the Kiddie Schedule for Affective Disorders and Schizophrenia for School-Age Children, Present and Lifetime Version (K-SADS-PL) (77) (for participants <16 years of age). Inclusion criteria across groups required participants to be in good general physical health, fluent in the English language, and within an age range of 12–30 years. Additionally, participants were excluded if they met DSM-IV criteria for alcohol or substance dependence within the past year (excepting nicotine dependence), had a history of head injury resulting in loss of consciousness, any significant medical or neurological illness with possible effects on the central nervous system, or a first-degree relative with a psychotic illness diagnosis. Written informed consent was obtained from all study participants (or parental consent and participant assent for participants <18 years of age), under protocols approved by the Human Subjects Subcommittee of the VA Connecticut Healthcare System, Hartford Hospital’s Institutional Review Board, and the Human Investigations Committee of the Yale University School of Medicine.

### Multi-load working memory task description

Participants performed a WM task, the Sternberg Item Recognition Paradigm (SIRP) (56), which consisted of two-, three-, four-, five-, and six-item loads. More specifically, each block began with the task instruction “learn” appearing on the screen for 1.5 s, followed by a blank screen for 0.5 s. Participants then saw a list of letters presented serially, each for 1.5 s, with jittered inter-stimulus intervals [ISIs of 1, 1.5, or 2 s (*encode phase*)]. Next, a blank screen appeared, for a jittered duration of 3, 4, 5, 6, or 7 s (*maintenance phase*). Then the task instruction “recall” appeared on the screen for 1.5 s, followed by a blank screen for 0.5 s. Participants then saw a list of six “probe” letters presented serially, each for 1.5 s, with jittered ISIs of 0.5, 1, or 1.5 s (*probe phase*). At the end of each probe phase, the task instruction “rest” appeared on the screen for 1.5 s, followed by a blank screen for 2 s. Each task run consisted of two memoranda sets being presented, at each load, for a total of 10 blocks per run.

Participants were instructed to press one response button with their dominant hand index finger for probe targets (i.e., items that were in the memoranda set presented during the encode phase) and to press another response button with their middle finger for probe foils (i.e., items that were not in the memoranda set). For loads 3–6, 50% of the trials were targets and 50% were foils. In order to ensure equivalent trial numbers across loads, 33% of the trials were targets and 67% were foils for load 2. Behavioral responses were recorded via a fiber-optic response system. Behavioral dependent measures of interest were median reaction times (RT) for correct responses (in milliseconds) and performance accuracy defined as the overall percentage of probe items that were correctly responded to (i.e., targets and foils) for a given load.

### Neuroimaging acquisition and processing

Brain images were acquired at the Olin Neuropsychiatry Research Center on a Siemens Allegra 3 T magnet. Participants completed three functional runs of the SIRP task. Functional images were collected in the axial plane with the following echoplanar sequence: TR = 1.5 s, TE = 27 ms, flip angle = 60°, FOV = 22 cm, 64 × 64 matrix, for 29 4 mm slices and a 1 mm inter-slice gap. Acquired voxel dimensions were 3.44 mm × 3.44 mm × 5 mm. To mitigate non-equilibrium effects, images corresponding to the first four TRs of each run were discarded from analysis.

Image processing was performed with Statistical Parametric Mapping (SPM8)^1^. Image preprocessing entailed motion correction (INRIAlign)^2^ via affine registration of all runs, where the first image of each run was realigned to the first image of the first run, and then re-alignment proceeded within each run. Images were then slice-time corrected. The Artifact Detection Tools (ART) toolbox^3^ was then used to identify outlying volumes in the time-series based on global image intensity values (>*Z* = 3) and head motion (>2 mm translational movement in *x*, *y*, or *z* plane or >0.02°rotation in yaw, pitch, or roll). ACompCor, a principal components-based approach to noise reduction of blood oxygen level-dependent (BOLD) data was then applied. ACompCor is based on deriving principal components of noise regions-of-interest (ROI), defined on white matter and CSF parcels from participants’ high-resolution anatomical images; these principal components are then used as nuisance regressors in the first-level modeling of the fMRI data to decrease the influence of noise and improve signal detection (78). Data were then spatially smoothed with a 6 mm FWHM Gaussian filter.

For individual participant (first-level) analyses, SPM’s canonical hemodynamic response function (a double gamma function, with a temporal derivative term) was convolved with task-vectors representing each of the three task phases (encode, maintain, probe) at each WM load level (2, 3, 4, 5, 6), yielding first-level task regressors. Seven motion parameters, calculated via the ART toolbox, consisting of the temporal derivatives of the six motion parameters as well as a summary measure of total motion, were included as regressors to remove fluctuations in BOLD signal attributable to participant head movement. To further ensure that the data were optimally cleaned of noise, regressors were also included for (i) data points identified by the ART toolbox as outliers and (ii) statistically significant (*p* < 0.05) principal noise components from the ACompCor denoising routine retained from each individual fMRI run based on a Monte Carlo simulation procedure (78). Regressors coding for each of the three fMRI runs were also included. Parameters (i.e., beta coefficients) representing the fit of each regressor to a voxel’s time series were estimated using the general linear model after applying a high pass temporal filter (128 s cut-off) to remove low-frequency noise. Mean functional images were normalized to a standard neuroanatomical space (Montreal Neurological Institute’s MNI-152 template), resulting in 3 mm^3^ isotropic voxel dimensions, and the normalization parameters were applied to first-level beta and contrast images. Group-based (second-level) analyses were then conducted on beta or contrast images, as described below in the data analysis plan.

To simplify analyses while simultaneously considering optimally distinct WM loads, analyses considered loads 2 (low), 4 (medium), and 6 (high) to address hypotheses related to WM load-related DMN suppression and DLPFC activation.

### Age-adjustment *Z*-scoring procedure

Given the ongoing neuromaturation expected during the age range studied (12–30 years), as well as the fact that the three groups differed in age (ESZ > HC > CHR), it was necessary to account for fMRI signal variance attributable to normal development in our analyses. We sought to remove only normal aging effects while preserving any variance associated with pathological age-related processes in the patient groups. Our approach, described previously (79–81), involves using the HC group to model normal aging effects, followed by calculation of age-adjusted *z*-scores for all subjects based on the HC group age-regression. Individual subject *z*-score maps for each fMRI beta or contrast image of interest were calculated as follows. First, normal aging effects were modeled by conducting a voxelwise regression of each fMRI beta or contrast map on age within the HC group. Second, for each voxelwise age regression, the HC age-regression equation was saved for use in generating predicted values for a healthy individual of a given age, and the standard error of regression was saved for use in estimating the standard deviation of beta or contrast values relative to the HC age-regression line. Third, for each beta or contrast image, and for each participant irrespective of group membership, an age-adjusted *z*-score was generated for each image voxel using the following calculation.

Age-adjusted *z*-score = (*observed beta or contrast value – beta or contrast value predicted for a healthy individual of a given age)/standard error of the HC age regression*.

Thus, an individual participant’s age-adjusted *z*-score voxelwise map reflects the deviation in brain activity, expressed in standard deviation units, from that expected for a healthy individual of a similar age. Behavioral data (median response time and performance accuracy) were adjusted for age using the same regression-based procedures.

### ROI definition

In order to address study hypotheses predicting load-dependent group differences in suppression of DMN activity, four anatomical ROI were selected, *a priori*. The four DMN regions examined were the mPFC, posterior cingulate cortex (PCC), left lateral posterior parietal cortex (lPPC), and right lateral posterior parietal cortex (rPPC). These regions were selected based on their consistent inclusion in the DMN in prior literature (9, 10, 18, 82).

The four DMN ROIs were defined functionally based on resting-state fMRI data collected from an independent sample of adolescent and young adult HCs with a similar mean age to the study sample (*n* = 28; mean age = 22.14 years). A seed-based connectivity analysis of the DMN was conducted using the CONN toolbox (83). One of the standard DMN ROIs available within the toolbox was used as the seed region to generate the connectivity analysis, and was defined by a 10 mm radius around a PCC coordinate (MNI −6*x*, −52*y*, 40*z* mm) originally employed in the DMN literature by Fox et al. (18). The PCC was chosen, *a priori*, as the seed for the connectivity analysis based on this region’s definition in prior literature as a major node of the DMN (5, 9, 10, 18, 82). After undergoing the same fMRI data preprocessing pipeline outlined above, a time series from the PCC seed was extracted for each participant in the independent HC sample, and voxelwise correlation maps were generated representing the Pearson’s *r* correlation value between the time series of the ROI seed and every voxel in the brain. These *r* values were transformed to *z*-scores via Fisher’s transformation, which were then averaged across individuals to produce a mean HC map representing functional connectivity with respect to the PCC ROI seed. This connectivity map was thresholded at a stringent height threshold (*p* = 1 × 10^−7^, uncorrected) in order to optimally isolate the four DMN ROIs (PCC, mPFC, lPPC, rPPC), which were then each saved as binary masks. These binary masks were further refined functionally through intersection with the union of each group’s thresholded (*p* < 0.001) deactivation maps (implicit baseline > probe activation) across probe levels.

In order to address study aims evaluating group differences in WM load-dependent DLPFC activation, left and right BA 46 ROIs were defined anatomically using the Talairach–Daemon-based Wake Forest University PickAtlas (84). These binary masks were further refined functionally through intersection with the union of each group’s thresholded (*p* < 0.001) activation maps (probe activation > implicit baseline) across probe levels.

For each participant, mean age-adjusted *z*-scores were then extracted via MATLAB from the intersected DMN and DLPFC ROIs in MNI space for low (two-item), medium (four-item), and high (six-item) probe conditions. These *z*-scores were imported into SPSS and subjected to Group-by-Load repeated-measures ANOVA (rmANOVA) analyses as described below.

### Data analysis plan

Age-adjusted WM task performance (median response time and mean probe accuracy) and fMRI data for the four DMN and two DLPFC ROIs were analyzed with separate rmANOVA models with Group as the between-subjects factor and Load (two-item, four-item, six-item) as the within-subjects factor. Significant Group-by-Load effects were followed up at each level of Load. Significant main effects of Group observed in the presence of a non-significant interaction were followed up by collapsing across Load. To control Type I error rate, significant omnibus effects (*p* < 0.05) were followed up with Tukey–Kramer HSD pairwise *post hoc* tests, to determine which groups contributed to observed omnibus effects. In addition to omnibus *F*-tests on main effects and interactions, polynomial contrasts were examined in order to specifically determine whether there were linear or quadratic load-dependent effects on task-related activations and deactivations. Previous studies have documented load-related changes in DLPFC function, including linear slopes as well as negative quadratic (i.e., “inverted-*U*”) relationships reflecting load effects that rise to a peak, then fall off once optimum WM capacity has been exceeded [for example, see Ref. (57, 85)]. Lastly, regression models examined the relationship between task performance and fMRI data, including evaluation of whether regression line slopes differed between the groups, and the relationship between symptom severity and fMRI activity was examined within CHR and ESZ groups via Pearson product-moment correlation coefficients.

In order to ensure that analysis of brain function was only conducted on participants who exhibited adequate task engagement, performance criteria were set requiring at least 50% accuracy for both low (two-item) and medium (four-item) WM loads in order to be included in the data analyses. Application of these minimum performance criteria led to exclusion of two HC, zero CHR, and two ESZ participants from the initially recruited sample whose data were not included in this study.

## Results

### Demographic and behavioral performance data

Mean group demographic characteristics, clinical symptom ratings, and statistical tests of group differences are reported in Table 1. As expected based on the illness course of schizophrenia, the CHR group was significantly younger than the ESZ group (*p* < 0.001), with the HC group showing an intermediary mean age. Between-group age differences were addressed by using age-adjusted *z*-scores for the analysis of task performance and fMRI dependent variables, as described in the Section “Materials and Methods.” Across the three participant groups, there were no differences in socioeconomic status (SES) as assessed with the Hollingshead Index (86), handedness as assessed with the Edinburgh inventory (87), or gender (*p* > 0.05). In addition, there were no group differences on mean number of artifact regressors or on the mean composite motion measure, reflecting similar image motion/quality assurance metrics across participant groups (*p* > 0.05).

**Table 1:** **Demographic, image quality, and clinical data for participants in the healthy control (HC), clinical high risk for psychosis (CHR), and early illness schizophrenia (ESZ) groups**.

	HC	CHR	ESZ
*n*°	54	32	22
Gender (% male)	63.0	65.6	72.7

	**Mean ± SD**	**Mean ± SD**	**Mean ± SD**

Age (years)*	19.5 ± 4.3	17.0 ± 3.4	22.1 ± 3.5
SES^∞^	50.7 ± 21.4	60.9 ± 17.2	55.9 ± 14.1
Handedness (% right-handed)	87.8	83.3	95.5
Image quality-mean number of artifact regressors	8.3 ± 9.1	11.7 ± 7.3	9.7 ± 9.5
Image quality-mean composite motion correction	0.21 ± 0.19	0.25 ± 0.14	0.24 ± 0.23
**Mean Symptom Ratings for Patient Groups**			
SOPS^§^ positive	–	2.3 ± 0.9	_
SOPS negative	–	1.9 ± 1.1	–
SOPS disorganized	–	1.4 ± 1.0	–
SOPS general	–	2.1 ± 1.0	–
PANSS^§^ positive	–	–	2.3 ± 0.6
PANSS negative	–	–	2.4 ± 0.7
PANSS general	–	–	2.1 ± 0.4

*°SES scores not available for 14 HC; 2 CHR; 0 ESZ; handedness scores not available for 5 HC, 2 CHR, 0 ESZ; Clinical ratings not available for 2 CHR participants and 2 ESZ participants*.

*^∞^Participant SES (socioeconomic status) measured by the Hollingshead 2-Factor Index. Higher Hollingshead scores indicate lower SES. All other assessment measures are scaled such that higher scores reflect greater levels of the measured variable*.

*^§^Clinical symptom mean ratings are derived from the SOPS for the CHR group and from the PANSS for the ESZ group*.

*Significant omnibus test, p < 0.05: *age: F(2, 105) = 11.38, p < 0.001; Tukey–Kramer HSD post hoc tests HC > CHR, p = 0.01; HC < ESZ, p = 0.02; CHR < ESZ, p < 0.001.

*Non-significant (p > 0.05) comparisons: gender: χ^2^(2, N = 108) = 0.66, p = 0.72; SES: F(2, 89) = 2.63, p = 0.08; Tukey–Kramer HSD post hoc tests HC < CHR, p = 0.06; Handedness: χ^2^(2, N = 101) = 1.79, p = 0.41*.

*Image quality-mean number of artifact regressors: F(2, 105) = 1.54, p = 0.22*.

*Image quality-mean composite motion correction: F(2, 105) = 0.47, p = 0.63*.

**Figure 1:**
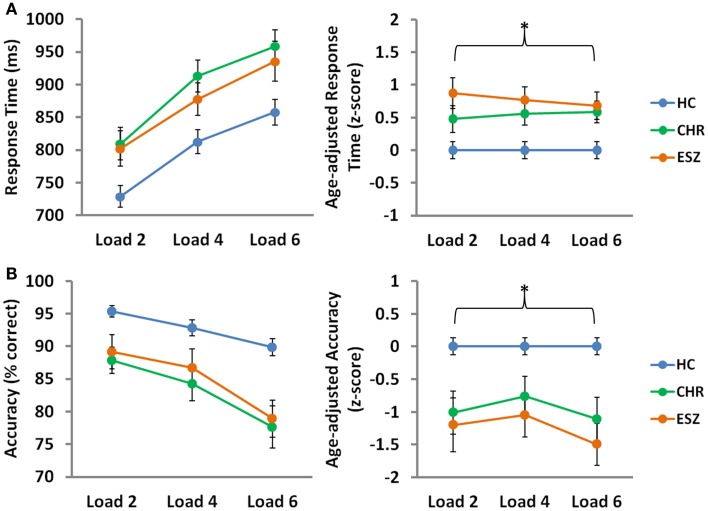
**Behavioral Data by Group for low (two-item), medium (four-item), and high (six-item) working memory loads during the probe condition. HC = healthy control; CHR = clinical high-risk; ESZ = early schizophrenia. Median response time (±standard error) is shown in the top panel and mean accuracy (±standard error) is shown in the bottom panel by Group, for unadjusted (left) and age-adjusted (right) data**.° °Statistical tests of behavioral data (*p < 0.05): Unadjusted behavioral data: *Median response time, main effect of load: *F*(2, 210) = 111.86, *p* < 0.001 *Mean accuracy, main effect of load: *F*(2, 210) = 39.49, *p* < 0.001 Age-adjusted behavioral data: data were adjusted for participant age via a *z*-scoring procedure based on an age regression within the HC group to model normal aging effects. As a result, the age-adjusted *z*-scores in the HC group have a mean = 0; SD = 1, and the means in the patient groups reflect the degree and direction of abnormality, in standard units, from the HC-derived age-specific norms. *Median response time, main effect of group: *F*(2, 105) = 6.84, *p* = 0.002; Tukey–Kramer HSD *post hoc* tests, HC < CHR, *p* = 0.03; HC < ESZ, *p* = 0.004 Median response time, interaction effect of group-by-load: *F*(4, 210) = 0.61, *p* = 0.66 *Mean accuracy, main effect of group: *F*(2, 105) = 10.27, *p* < 0.001; Tukey–Kramer HSD *post hoc* tests, HC > CHR, *p* = 0.003; HC > ESZ, *p* < 0.001 Mean accuracy, interaction effect of group-by-load: *F*(4, 210) = 0.79, *p* = 0.53.

Main effects of load, collapsed across the three groups, were observed for median response time (*p* < 0.001) and mean probe accuracy (*p* < 0.001), indicating that task performance decreased with cognitive load, as expected by the parametric task design (56). To compare performance across groups on the SIRP WM fMRI task, Group-by-Load rmANOVA models were run on median response time and mean accuracy age-corrected *z*-scores. Analysis of median response time *z*-scores revealed a significant main effect of Group (*p* = 0.002), driven by slower response latencies in both CHR (*p* = 0.03) and ESZ (*p* = 0.004) groups, relative to HC. Similarly, a significant main effect of Group (*p* < 0.001) was observed for accuracy scores, and was explained by lower accuracy in both CHR (*p* = 0.003) and ESZ (*p* < 0.001) groups, relative to HC. Group-by-Load interaction effects were non-significant for both response time and accuracy *z*-score measures (*p* > 0.05), indicating that the poorer performance evidenced by both patient groups, relative to HC, did not significantly depend on load. Behavioral performance data and results of statistical tests of group differences are shown in Figure 1.

### fMRI data

#### Default mode network analysis

**Figure 2:**
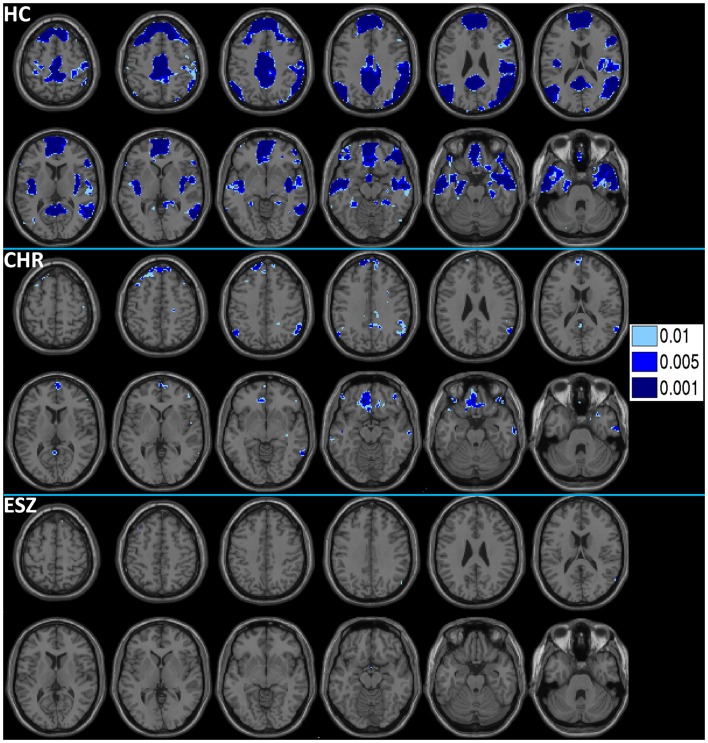
**Deactivations for the within-group (HC = healthy control; CHR = clinical high-risk; ESZ = early schizophrenia) linear contrast of working memory loads during the probe condition (high > medium > low loads)**. Regions of greater deactivation with increasing load are shown in cool colors at three uncorrected height thresholds (*p* < 0.01, *p* < 0.005, and *p* < 0.001), and include default mode network (DMN) nodes such as the medial prefrontal cortex, posterior cingulate cortex, and right and left lateral posterior parietal cortices in HC participants. Extent of axial montage is 58 mm > *Z* > −30 mm, with a 8-mm skip.

**Figure 3:**
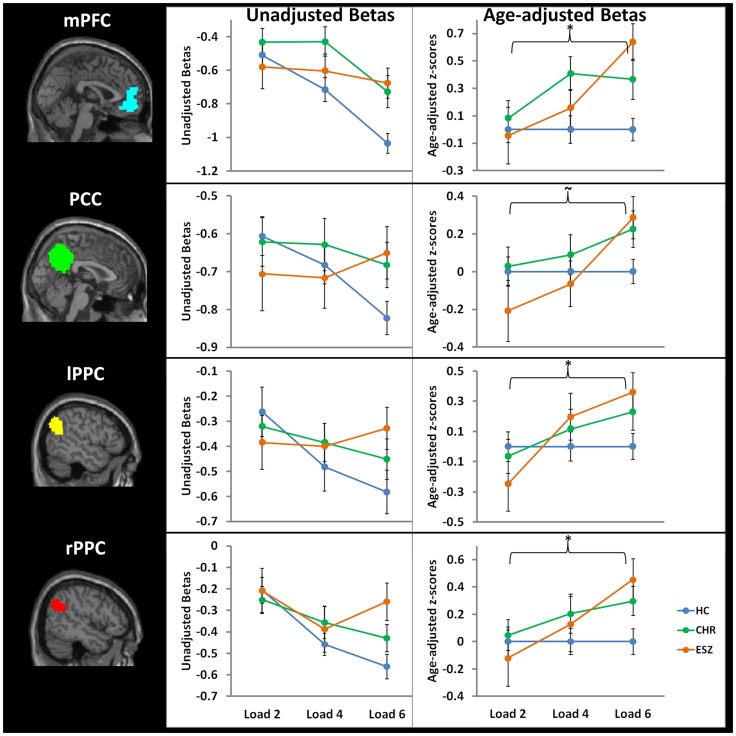
**Four default mode network regions-of-interest (ROIs) were interrogated for group-by-load interaction effects: medial prefrontal cortex (mPFC), posterior cingulate cortex (PCC), left lateral posterior parietal cortex (lPPC), and right lateral posterior parietal cortex (rPPC). Line graphs display each ROI’s mean fMRI contrast value (±standard error) by Group, for low (two-item), medium (four-item), and high (six-item) working memory loads during the probe condition. Unadjusted (left) and age-adjusted (right) data are shown in figure panels**.° °Statistical tests of age-adjusted extracted fMRI data (*p < 0.05; ∼0.08 < *p* < 0.05): data were adjusted for participant age via a *z*-scoring procedure based on an age regression within the HC group to model normal aging effects. As a result, for each voxel in the brain, the age-adjusted *z*-scores in the HC group have a Mean = 0; SD = 1, and the means in the patient groups reflect the degree and direction of abnormality, in standard units, from the HC-derived age-specific norms. *rmANOVA medial prefrontal cortex ROI, main effect of group: *F*(2, 105) = 4.35, *p* = 0.02 *rmANOVA medial prefrontal cortex ROI, interaction effect of group-by-load: *F*(4, 210) = 2.69, *p* = 0.03
One-way ANOVA at load 2, main effect of group: *F* (2, 105) = 0.20, *p* = 0.82*One-way ANOVA at load 4, main effect of group: *F*(2, 105) = 3.36, *p* = 0.04; Tukey–Kramer HSD *post hoc* tests, HC < CHR, *p* = 0.03*One-way ANOVA at load 6, main effect of group: *F*(2, 105) = 7.57, *p* = 0.001; Tukey–Kramer HSD *post hoc* tests, HC < CHR, *p* = 0.05; HC < ESZ, *p* = 0.001 rmANOVA posterior cingulate cortex ROI, main effect of group: *F*(2, 105) = 0.83, *p* = 0.44 ∼rmANOVA posterior cingulate cortex ROI, interaction effect of group-by-load: *F*(4, 210) = 2.36, *p* = 0.06
One-way ANOVA at load 2, main effect of group: *F*(2, 105) = 1.13, *p* = 0.33One-way ANOVA at load 4, main effect of group: *F*(2, 105) = 0.51, *p* = 0.60*One-way ANOVA at load 6, main effect of group: *F*(2, 105) = 3.38, *p* = 0.04; Tukey–Kramer HSD *post hoc* tests, HC < ESZ, *p* = 0.07 rmANOVA right lateral posterior parietal cortex ROI, main effect of group: *F*(2, 105) = 1.04, *p* = 0.36 *rmANOVA right lateral posterior parietal cortex ROI, interaction effect of group-by-load: *F*(4, 210) = 2.50, *p* = 0.04
One-way ANOVA at load 2, main effect of group: *F*(2, 105) = 0.31, *p* = 0.73One-way ANOVA at load 4, main effect of group: *F*(2, 105) = 0.70, *p* = 0.50*One-way ANOVA at load 6, main effect of group: *F*(2, 105) = 4.23, *p* = 0.02; Tukey–Kramer HSD *post hoc* tests, HC < ESZ, *p* = 0.02 rmANOVA left lateral posterior parietal cortex ROI, main effect of group: *F*(2, 105) = 0.42, *p* = 0.66 *rmANOVA left lateral posterior parietal cortex ROI, interaction effect of group-by-load: *F*(4, 210) = 3.05, *p* = 0.02
One-way ANOVA at load 2, main effect of group: *F*(2, 105) = 0.90, *p* = 0.41One-way ANOVA at load 4, main effect of group: *F*(2, 105) = 0.66, *p* = 0.52∼One-way ANOVA at load 6, main effect of group: *F*(2, 105) = 2.84, *p* = 0.06; Tukey–Kramer HSD *post hoc* tests, HC < ESZ, *p* = 0.08

Prior to examining probe Group-by-Load interactions for DMN ROIs, we inspected the voxelwise contrast maps (at thresholds of *p* < 0.01, *p* < 0.005, and *p* < 0.001, uncorrected) showing the linear effect of load during the WM probe period in the HC group to determine whether the expected pattern of greater DMN deactivation with increasing load was present (see Figure 2, top panel). HC showed prominent linear load-dependent deactivations in DMN regions consisting of the mPFC (including bilateral BA 10/11, and medial BA 9), PCC (including BA 30/31, precuneus), and right and left lateral posterior parietal cortices (including angular gyri, BA 39/40). When we examined this voxelwise linear contrast map within the CHR and ESZ groups, load-dependent DMN deactivation during the WM probe period was much less evident, particularly at higher significance thresholds (see Figure 2, middle and bottom panels). In order to evaluate study hypotheses, mean beta values unadjusted for age and age-corrected *z*-scores were extracted from the DMN ROIs for the low, medium, and high WM load probe periods. Unadjusted betas were examined to assess within-group load-dependent deactivation patterns, while age-corrected *z*-scores were examined to assess between-group differences using Group-by-Load rmANOVA. Significant omnibus effects (*p* < 0.05) were followed up as described in the data analysis plan. Mean unadjusted beta values and age-corrected *z*-scores for each probe load level are shown for each Group in Figure 3. In addition, test statistics, including omnibus rmANOVA models and indicated follow-up tests, are reported in Figure 3. Non-significant trends (0.08 > *p* > 0.05) were also reported in order to determine which groups contributed to significant omnibus Group and Group-by-Load effects emerging from the rmANOVA.

We first analyzed the mean unadjusted beta values across loads for each DMN ROI within each group to characterize load-dependent patterns of functional deactivation during the WM probe period (see Figure 3, left panel). Two single degree of freedom polynomial contrasts (linear, quadratic) across loads were examined within each group. For the mPFC, both the HC (*p* < 0.001) and CHR (*p* = 0.02) groups showed a significant linear increase in deactivation from low to high WM loads, while the ESZ group did not show this pattern (*p* = 0.58). For the PCC, there was a strong linear increase in deactivation with increasing WM load in the HC group (*p* < 0.001) but not in the CHR (*p* = 0.41) or ESZ (*p* = 0.66) groups. For the lPPC, there was a strong linear increase in deactivation with increasing WM load in the HC group (*p* < 0.001) that was attenuated and non-significant in the CHR group (*p* = 0.10) and not evident in the ESZ group (*p* = 0.66). Within the rPPC, there was a linear increase in deactivation with increasing WM load in both the HC (*p* < 0.001) and CHR (*p* = 0.02) groups that was not present in the ESZ group (*p* = 0.61). Quadratic contrasts were non-significant within the HC (0.51 > *p* > 0.11) and CHR (0.99 > *p* > 0.09) groups across all four DMN regions examined. Within the ESZ group, the quadratic contrasts were similarly non-significant for mPFC (*p* = 0.82), PCC (*p* = 0.49), and lPPC (*p* = 0.62); however, there was a significant quadratic pattern of deactivation within the rPPC (*p* = 0.03) involving greatest deactivation at load 4 with relatively smaller deactivations at loads 2 and 6.

Direct comparisons between the groups, controlling for normal aging effects, were performed using age-corrected *z*-scores in Group-by-Load rmANOVAs for each DMN ROI (see Figure 3, right panel). Results for each DMN ROI are as follows.

##### Medial prefrontal cortex

For the mPFC, there was a significant main effect of Group (*p* = 0.02) and a significant Group-by-Load interaction (*p* = 0.03). Focusing on specific polynomial contrasts to examine linear and quadratic trends across loads, there was a significant Group-by-Linear effect (*p* = 0.03) but the Group-by-Quadratic effect was not significant (*p* = 0.26). The linear effect was explained by ESZ showing a significantly worsening suppression deficiency (i.e., abnormally large *z*-score) with increasing load relative to the flat “normative” *z*-score profile across loads in the HC group (*p* = 0.03). Follow-up one-way ANOVAs examining the Group effect at each Load revealed significant Group effects for load 4 (*p* = 0.04) and load 6 (*p* = 0.001). Tukey–Kramer HSD *post hoc* tests indicated that the CHR group showed significantly less mPFC deactivation than HC at both load 4 (*p* = 0.03) and load 6 (*p* = 0.05). ESZ also showed significantly less mPFC deactivation than HC at load 6 (*p* = 0.001).

##### Posterior cingulate cortex

For the PCC, while the Group effect was not significant (*p* = 0.44), there was a trend toward a significant Group-by-Load interaction (*p* = 0.06). Polynomial contrast analysis indicated a significant Group-by-Linear effect (*p* = 0.02) but no Group-by-Quadratic (*p* = 0.75) effect. The linear effect was explained by ESZ showing a significantly worsening suppression deficiency with increasing load (*p* = 0.02). Follow-up one-way ANOVAs at each Load indicated a significant Group effect at Load 6 (*p* = 0.04), with pairwise Tukey–Kramer HSD *post hoc* tests indicating a trend (*p* = 0.07) for ESZ to deactivate this region less than HC.

##### Left lateral posterior parietal cortex

For the lPPC, while there was not a significant Group effect (*p* = 0.66), there was a significant Group-by-Load interaction (*p* = 0.02). Polynomial contrast analysis indicated a significant Group-by-Linear effect (*p* = 0.005) with no Group-by-Quadratic (*p* = 0.67) effect. The linear effect was explained by ESZ showing a significantly worsening suppression deficiency with increasing load (*p* = 0.004). Follow-up one-way ANOVAs at each Load indicated no significant main effects of Group, though a trend toward significance was observed at load 6 (*p* = 0.06) with *post hoc* tests indicating a trend (*p* = 0.08) toward deficient deactivation in the ESZ relative to the HC, similar to the pattern observed in the PCC.

##### Right lateral posterior parietal cortex

For the rPPC, the main effect of Group was not significant (*p* = 0.36) but there was a significant group-by-load interaction (*p* = 0.04). Polynomial contrast analysis indicated a significant Group-by-Linear effect (*p* = 0.01) with no Group-by-Quadratic (*p* = 0.92) effect. Similar to the other DMN ROIs, the linear effect was explained by ESZ showing a significantly worsening suppression deficiency with increasing load (*p* = 0.008). Follow-up one-way ANOVAs at each Load indicated a significant Group effect at Load 6 (*p* = 0.02), with pairwise *post hoc* tests indicating that ESZ deactivated rPPC significantly less than HC (*p* = 0.02).

#### Dorsolateral prefrontal cortex analysis

**Figure 4:**
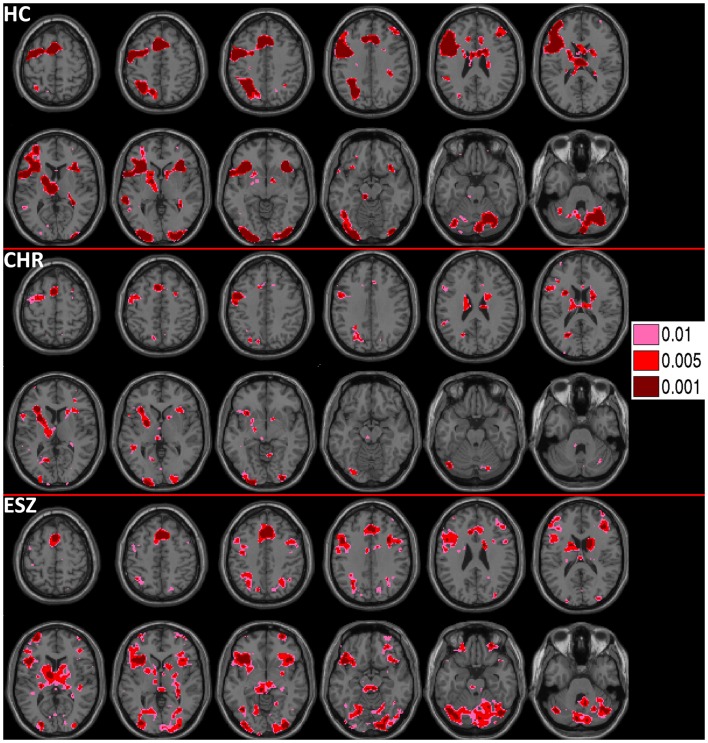
**Activations for the within-group (HC = healthy control; CHR = clinical high-risk; ESZ = early schizophrenia) linear contrast of working memory loads during the probe condition (high > medium > low loads)**. Regions of greater activation with increasing load are shown in hot colors at three uncorrected height thresholds (*p* < 0.01, *p* < 0.005, and *p* < 0.001), and include left dorsolateral prefrontal cortex and dorsal anterior cingulate cortex. Extent of axial montage is 58 mm > *Z* > −30 mm, with an 8-mm skip.

**Figure 5:**
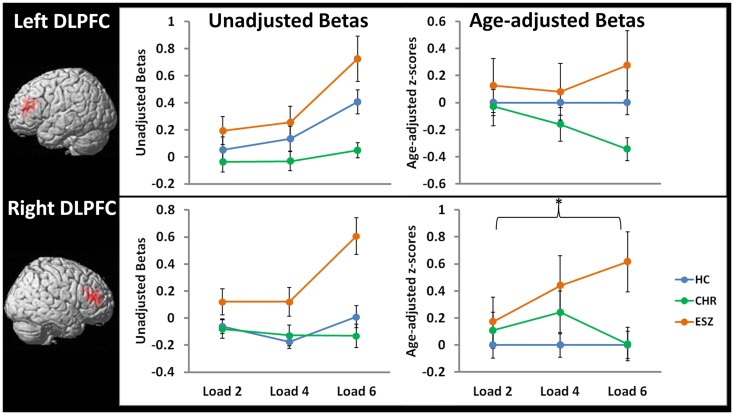
**Line graphs display mean fMRI contrast value (±standard error) for left and right DLPFC regions-of-interest by Group, for low (two-item), medium (four-item), and high (six-item) working memory loads during the probe condition. Unadjusted (left) and age-adjusted (right) data are shown in figure panels**.° °Statistical tests of age-adjusted extracted fMRI data (*p < 0.05): data were adjusted for participant age via a *z*-scoring procedure based on an age regression within the HC group to model normal aging effects. As a result, for each voxel in the brain, the age-adjusted *z*-scores in the HC group have a mean = 0; SD = 1, and the means in the patient groups reflect the degree and direction of abnormality, in standard units, from the HC-derived age-specific norms. rmANOVA left DLPFC ROI, main effect of group: *F*(2, 105) = 2.10, *p* = 0.13 rmANOVA left DLPFC ROI, interaction effect of group-by-load: *F*(4, 210) = 1.37, *p* = 0.25 *rmANOVA right DLPFC ROI, main effect of group: *F*(2, 105) = 3.58, *p* = 0.03; Tukey–Kramer HSD *post hoc* tests, collapsed across load, HC < ESZ, *p* = 0.02 rmANOVA right DLPFC ROI, interaction effect of group-by-load: *F*(4, 210) = 1.64, *p* = 0.17

Prior to examining probe Group-by-Load interactions, we inspected the voxelwise contrast maps for the linear effect of load during the WM probe period within each group. Whole brain maps of these linear load effects within each group are shown in Figure 4 for thresholds of *p* < 0.01, *p* < 0.005, and *p* < 0.001, uncorrected. Inspection of Figure 4 indicates that HC showed a linear increase in activation with increasing WM load in regions expected based on prior literature (88–90) including the DLPFC, inferior parietal lobe, and anterior cingulate cortex. To further examine these load effects, mean beta values, unadjusted for age, were extracted from left and right DLPFC ROIs and subjected to a one-way rmANOVA within each participant group to characterize load-dependent trajectories of WM probe activation in the DLPFC (see Figure 5, left panel). Two single degree of freedom polynomial contrasts (linear, quadratic) were evaluated for significant (*p* < 0.05) effects within each group. For the left DLPFC, the HC group showed a highly significant linear increase in activation with load (*p* < 0.001), along with a non-significant trend toward a quadratic increase (*p* = 0.09). The CHR group showed peri-baseline activation in this region that varied little across loads reflected by non-significant linear (*p* = 0.19) and quadratic (*p* = 0.65) effects. The ESZ group showed significant linear (*p* = 0.007) and quadratic (*p* = 0.001) effects indicating load dependent increases in DLPFC activation, particularly at the highest load. For the right DLPFC, the HC group showed a load-dependent pattern of brain activation consisting of peri-baseline activity at the lowest load, modest deactivation at the middle load, and modest activation at the highest load (linear contrast, *p* = 0.28; quadratic contrast, *p* = 0.008). The CHR group showed peri-baseline activation in this region that varied little across loads reflected by non-significant linear (*p* = 0.53) and quadratic (*p* = 0.82) effects. In contrast, the ESZ group was the only group to activate the right DLPFC ROI across all three loads, showing increased activation with load as reflected by significant linear (*p* = 0.004) and quadratic (*p* = 0.02) effects.

In order to directly compare the groups while controlling for normal aging, mean age-adjusted *z*-scores were extracted from the right and left DLPFC ROIs and subjected to Group-by-Load rmANOVA (see Figure 5, right panel). For the lDLPFC, neither a significant main effect of Group (*p* = 0.13) nor a Group-by-Load interaction effect (*p* = 0.25) was observed. Similarly, Group-by-Linear (*p* = 0.12) and Group-by-Quadratic (*p* = 0.70) interactions were non-significant. For the rDLPFC, a significant main effect of Group was observed (*p* = 0.03) that did not significantly interact with load (*p* = 0.17). Polynomial contrast analysis indicated a significant Group-by-Linear effect (*p* = 0.04) with no Group-by-Quadratic (*p* = 0.61) effect. The linear effect was explained by ESZ showing a significant increase in rDLPFC age-adjusted *z*-scores relative to CHR (*p* = 0.05) and with a statistical trend toward an increase relative to HC (*p* = 0.08). Tukey–Kramer HSD-corrected follow-up comparisons, collapsed across load, indicated that the main effect of Group was explained by ESZ activating right DLPFC more than HC (*p* = 0.02), with the CHR group differing from neither group. See Figure 5 for plots of DLPFC ROI means by Group and test statistics.

#### Correlation of fMRI activity with symptom severity

We next considered the relationship between symptom severity and WM task-related activations and deactivations for each ROI, separately within the CHR and ESZ groups. Specifically, beta values reflecting the WM probe effect were averaged within the DMN and DLPFC ROIs for each subject at each WM load and correlated with each subject’s mean positive and negative symptom severity scores (measured by the SOPS for CHR participants and the PANSS for ESZ participants). Within the CHR group, significant associations were detected between negative symptom severity and DMN deactivations within the rPPC at Load 2 (*r* = −0.38; *p* = 0.02) and Load 4 (*r* = −0.40; *p* = −0.03), and within the PCC at Load 4 (*r* = −0.36, *p* = 0.05). Within the CHR group significant associations were also detected between positive symptom severity and DMN deactivations within the lPPC at Load 4 (*r* = −0.38; *p* = 0.04), and within the PCC at Load 2 (*r* = −0.38; *p* = 0.04) and Load 6 (*r* = −0.44; *p* = 0.02). There were no significant correlations observed within the ESZ group between positive or negative symptom severity and fMRI activity within any of the ROIs examined (0.93 > *p* > 0.15).

#### Correlation of load-dependent fMRI activations with task performance

Lastly, we considered the relationship between WM probe-related regional brain activity and task performance at each WM load. Specifically, beta values reflecting the WM probe effect were averaged within the DMN and DLPFC ROIs for each subject at each WM load and regressed on Group and load-specific task Performance at Step 1 of the hierarchical regression model. At Step 2, the Group-by-Performance interaction was entered, testing for significant group differences in the slopes of the regression lines relating fMRI activity to Performance. When these slope differences were not significant, the common slope estimated for the Performance measure for all groups was tested for significance. Performance measures included mean accuracy (percent correct) and response time to the WM probes for each load. None of the Group-by-Performance interaction effects were significant at Step 2 (Group-by-Accuracy 0.99 > *p* > 0.11; Group-by-RT 0.99 < *p* < 0.21), indicating equivalent slopes across groups for all relationships examined. Subsequent tests of the common slope for each performance measure and ROI revealed significant positive relationships between accuracy and DLPFC activation at Load 6 for both lDLPFC (*p* = 0.02) and rDLPFC (*p* = 0.01). In addition, several significant associations or trends were detected for response time to the WM probes. Slower response times were associated with greater DMN deactivations at Load 2 (lPPC, *p* = 0.02; rPPC, *p* = 0.01; PCC, *p* = 0.09) and at Load 6 (lPPC, *p* = 0.04; rPPC, *p* = 0.08). In contrast, greater lDLPFC activation was related to faster response times for Load 2 (*p* = 0.06) and Load 4 (*p* = 0.05). Of note, almost all of the significant Group differences in the DMN regions and rDLPFC reported above persisted as significant Group effects when controlling for accuracy in Step 1 of the hierarchical regression model (all *p* < 0.054). The exceptions were for the lPPC, where the Group effect controlling for accuracy at Load 6 remained a trend (*p* = 0.07), and the PCC, where the Group effect was no longer significant (*p* = 0.17) after controlling for accuracy at Load 6.

## Discussion

This study used fMRI to assess brain activity during a multi-load SIRP WM task in individuals at CHR for psychosis, ESZ patients, and HCs, with an emphasis on evaluating patterns of load-dependent deactivation in DMN regions. Results showed that CHR patients exhibited deficient suppression of DMN activity in the mPFC at moderate and high WM loads relative to HC participants. ESZ patients also exhibited deficits in DMN suppression at higher WM loads, but unlike the CHR patients, the deficits were evident across multiple nodes of the DMN including mPFC and rPPC, with similar trends in the lPPC and the PCC. In contrast to the DMN abnormalities, CHR patients exhibited normal DLPFC activation during WM retrieval, whereas ESZ patients showed a load-dependent increase in “inefficient” (i.e., hyperactive) right DLPFC activation relative to HC and CHR participants. In terms of task performance, all participants, irrespective of group, exhibited the expected load-dependent increase in response time and decrease in accuracy as WM load increased parametrically from two to six items (56). Examination of age-adjusted task performance data revealed slower and less accurate responses to WM probes across all loads in the CHR and ESZ patient groups, relative to the HC group.

With respect to DMN deactivation patterns, within-group analyses indicated that the HC group showed highly significant linear load-dependent increases in deactivation within all four of the *a priori* DMN regions examined while responding to the WM probes. When comparing groups across loads using age-adjusted *z*-scores, significant Group-by-Load interactions were observed in the mPFC, rPPC, and lPPC, and a non-significant trend (*p* = 0.06) toward a Group-by-Load effect was observed in the PCC. Follow-up testing indicated that within the rPPC, observed effects resulted from deficient suppression at the highest WM load in the ESZ group relative to the HC group, with the CHR patients differing from neither group. In PCC and lPPC there were trends toward deficient suppression at the highest WM load in ESZ relative to HC. In mPFC, both CHR and ESZ groups showed abnormally deficient suppression of activity at higher WM loads, relative to the HC group. Interestingly, while DMN suppression was not correlated with WM accuracy, greater suppression of the posterior nodes of the DMN (PPC and PCC) was associated with slower response times to the WM probes in all groups. At least two interpretations of this effect are possible. One possibility is that slower individuals require greater DMN suppression to perform the task. Alternatively, greater DMN suppression may be associated with strategic efforts to optimize accuracy by slowing response times to the WM probes. Within the CHR group, several correlations were observed indicating that greater positive and negative symptom severity was associated with more posterior node (PCC, rPPC, lPPC) DMN suppression across several WM loads. These findings demonstrate that despite an absence of group differences between CHR and HCs in deactivation of posterior nodes of the DMN, understanding DMN activity in these regions may be clinically relevant through association with symptom severity. Though speculative, observed correlations could indicate that the more symptomatic CHR individuals needed to engage greater DMN suppression in order to perform the task, relative to their less symptomatic peers. Such a compensatory strategy may break down or become inadequate as illness progresses or symptom severity increases, which could explain the lack of observed symptom correlations in our ESZ participants. That we did not observe similar correlations with symptom severity in the ESZ group, therefore, may reflect substantive differences in the relationship between DMN suppression and symptom severity as a function of neurodevelopment or illness progression, or alternatively may reflect methodological differences, such as the different measures used to assess symptom severity between the two patient groups.

Our findings of altered DMN function integrate well with previous literature on DMN activity and connectivity in schizophrenia (4). Specifically, the present study’s finding of deficient suppression of DMN region activity in the ESZ group, particularly at high WM loads, is consistent with several prior reports and adds to a growing literature suggesting a hyperactive DMN in schizophrenia (21, 24, 25, 27, 91), though it should be noted that hypoconnectivity within the DMN has been reported as well [for example, see Ref. (22) and (30)]. Importantly, deficient DMN suppression has been shown to persist when schizophrenia patients and HC are performance-matched (24, 25, 27), suggesting that performance differences do not account for the group difference in DMN suppression. Similarly, in the current study, the majority of the group differences in DMN suppression persisted after controlling for task accuracy. Prior research has demonstrated that in the context of goal-directed tasks, the reciprocal relationship between task-positive and DMN activity present in HC, breaks down in patients with schizophrenia (27, 91). Further, it has been suggested that a lack of optimal DMN suppression during cognitive task engagement may be an independent source of cognitive impairment in schizophrenia beyond dysfunction of task-specific substrates (3). The typically anti-correlated relationship between the DMN and task-positive regions such as the DLPFC is reduced in patients with schizophrenia (25). This finding has been extended to the putative prodrome of schizophrenia (55) suggesting that disruptions of the resting-state interactions between DMN and task-positive circuits may predate psychosis onset. Such disruption of the normative interaction between DMN and task-positive circuits may result in impaired ability in schizophrenia to efficiently switch between intrinsic and extrinsic environmental demands (3, 4). Altered glutamatergic neurotransmission via NMDA receptor dysfunction has been proposed as a possible mechanism of inadequate DMN suppression in schizophrenia (92). This hypothesis is supported by empirical findings that administration of the NMDA receptor antagonist ketamine elicited dysfunctions of both fronto-parietal control region activations and DMN deactivations during WM in HC (92). Moreover, the DMN suppression induced by ketamine predicted poorer WM performance (92), underscoring the behavioral consequences of inadequate DMN suppression.

Functionally, DMN regions have primarily been implicated in governing internally focused cognitive processes, including autobiographical memory, theory of mind, moral judgment, and mental simulation, though an alternative body of literature suggests that the network may be involved in non-specific external monitoring functions (10). Within the DMN, mPFC function appears to support self-reflective cognitive processes, including social and emotional inference, and it has been proposed that mPFC is the hub of a DMN subsystem that subserves mental simulation functions in the service of socially or self-relevant reflection and planning (10). Our observation of deficient DMN suppression at higher WM loads in CHR individuals was restricted to the mPFC, suggesting that the self-reflective cognitive processes associated with mPFC activity are overactive in CHR individuals, particularly when these internally focused cognitive processes must be suppressed in order to attend to exogenous task demands. Thus, this deficient suppression of the mPFC node of the DMN may be among the earliest indications of DMN dysregulation in schizophrenia, being evident prior to psychosis onset in individuals with putatively prodromal symptoms.

In addition to DMN functional alterations, we identified WM-related DLPFC activations that differed by group. Within-group analysis of load-dependent DLPFC activations in the HC group revealed a linear increase in activation with load in the lDLPFC. While the HC group also showed a significant quadratic trend with load in the rDLPFC, this mainly reflected a slight deactivation at Load 4 and a small activation at Load 6, with the overall pattern suggesting very little activation of the rDLPFC across loads. Group comparisons of age-adjusted DLPFC activation *z*-scores across loads showed group differences that did not interact with load. Specifically, ESZ patients showed significantly greater rDLPFC activation than HC participants, suggesting inefficient recruitment of rDLPFC. Interestingly, while the lDLPFC appeared to be more activated than the right DLPFC in HC, especially at the highest load, group differences in the lDFLPC did not reach statistical significance in the overall ANOVA model. However, if we only consider the highest WM load, the lDLPFC showed inefficient activation in the ESZ relative to the deficient activation exhibited by CHR individuals (*p* = 0.01), with HC falling in between (see Figure 5). While it is possible that CHR individuals failed to activate DLPFC at the highest WM load because the load exceeded their WM capacity leading to disengagement from the task, this was not born out by the task performance data. Performance data showed CHR individuals to exhibit modest performance deficits that did not worsen with increasing load, and the deficits were comparable to those exhibited by the ESZ patients despite the fact that ESZ showed excessive DLPFC recruitment at the highest load. Overall, the pattern of results suggest that inefficient engagement of DLFPC during WM retrieval may develop after the onset of schizophrenia, with those at risk for psychosis either showing normal or somewhat deficient DLPFC activation during processing of higher WM loads.

The majority of prior fMRI WM studies in schizophrenia have studied adults with chronic illness. DLPFC dysfunction during WM and other executive control tasks is a hallmark finding in schizophrenia (1, 2, 57–62, 85, 93). Here, we extend findings of altered DLPFC function during WM to earlier stages of the illness course by showing inefficient (i.e., hyperactive) DLPFC activation, particularly at higher WM loads, in adolescent and young adult schizophrenia patients. Both hypoactivation and hyperactivation of PFC and more specifically of DLPFC have been reported in patients with schizophrenia relative to controls (57, 94), and hypotheses have been advanced to account for these seemingly discrepant findings. Differences in the point at which patients versus controls reach WM capacity with increasing WM load has been proposed as one possible factor contributing to whether schizophrenia-related DLPFC dysfunction manifests as increased or decreased fMRI activation relative to control participants (57). According to this theoretical perspective, at lower WM loads schizophrenia patients show inefficient PFC activity, activating more than HC. At higher loads, schizophrenia patients tend to reach their WM capacity sooner than HC, resulting in disengagement from the task and hypoactivation of DLPFC. Some studies have found evidence to support this theoretical framework [e.g., Ref. (91, 95)]. In the current study, task performance data showed that even at the highest WM load, patients performed well above chance, indicating that the challenge to WM was still well below their capacity. Indeed, it was at the highest WM load that ESZ patients appeared most inefficient in terms of DLPFC activation relative to HC and CHR participants. Across groups, greater fMRI activations of both lDLPFC and rDLPFC were associated with more accurate WM performance at Load 6 only. This suggests that coupling between DLPFC activation and performance emerged as WM demands increased, perhaps as a reflection of the greater resources needed to sustain task performance in the face of increasing processing demands. In addition, greater lDLPFC activation was related to faster response times across participant groups, suggesting that the magnitude of DLPFC engagement may contribute to the speed of WM retrieval.

The pattern of inefficient DLPFC activation observed in ESZ was not evident in our CHR sample. While some prior fMRI studies have documented normal DLPFC activation during WM performance in CHR patients (96, 97), consistent with our findings, at least one study using an *n*-back task found intermediate levels of DLPFC hypoactivation relative to the deficient activation observed in a recent onset schizophrenia group (98). In addition, studies have shown that the WM-related DLPFC impairments commonly observed in patients with schizophrenia are present, at least to some degree, in unaffected first-degree relatives of probands (99, 100). These data indicate a possible genetic contribution to WM-related brain dysfunction and vulnerability toward the disorder. The fact that we did not see these effects in CHR patients as a group may reflect the fact that CHR criteria do not necessarily identify individuals at genetic high risk for schizophrenia. Whether WM-related DLPFC dysfunction is present in the subgroup of CHR patients who go on to convert to psychosis remains to be determined. While this issue can be addressed as we obtain more clinical follow-up data from our sample, the limited data available, including ours, is noteworthy for failing to show any evidence of DLPFC inefficiency during WM performance in CHR patients. In addition, whereas CHR patients in our study exhibited task accuracy deficits relative to healthy subjects, task accuracy in CHR patients was normal in prior fMRI WM studies (96–98).

This study is limited by several factors. Foremost, our cross-sectional study design does not permit inferences about the impact of illness progression on DMN and DLPFC function within individuals during transition from CHR states to psychosis or during early years of schizophrenic illness. Further, we did not have access to sufficient clinical follow-up data to permit baseline comparisons of CHR patients who subsequently converted to a psychotic illness with those who did not convert. Thus, we cannot rule out the possibility that more extensive abnormalities may be evident in the subgroup of CHR patients who go on to convert to psychosis. Future studies employing longitudinal designs across the illness course from clinical risk to conversion, within individuals, would offer helpful extensions of this work. Despite these limitations, these data demonstrate that in the setting of increasing WM demand, antipsychotic naïve patients at clinical risk for psychosis show (i) task performance deficits indicating compromised WM function, (ii) deficient DMN deactivation at higher WM loads, particularly in the mPFC, that is similar to, but less extensive than, the deficient suppression exhibited by ESZ patients across DMN regions, and (iii) relatively preserved or slightly deficient DLPFC activation patterns during WM in sharp distinction from the inefficiency exhibited by ESZ patients at the highest WM load. Thus, the failure of normative DMN suppression during cognitive task performance that has previously been observed in chronic schizophrenia patients is present to a lesser extent during the putative prodromal stage of illness and may represent an earlier sign of impending psychosis than the DLPFC dysfunction that is a hallmark of schizophrenia. The DMN deficits in our CHR patients are consistent with the intermediate phenotype of brain dysfunction that has been described in the neuroimaging literature on CHR patients to date [reviewed by Ref. (63)]. Further research into the trajectories of brain development from putatively prodromal states through conversion to early illness is needed to determine whether the more extensive DMN and DLPFC dysfunction observed in schizophrenia patients relative to high-risk individuals reflects an intermediate level of abnormality that progresses during the transition to psychosis or a full-blown abnormality evident only in the subgroup of CHR patients who go on to convert to psychosis.

## Conflict of Interest Statement

This research was conducted in the absence of any commercial or financial relationships that could be construed as a potential conflict of interest. Dr. Mathalon has received consulting fees from Pfizer, Inc., and funding for an investigator-initiated study from AstraZeneca, Inc.

## Footnotes

^1^http://www.fil.ion.ucl.ac.uk/spm/software/spm8/

^2^http://www.fil.ion.ucl.ac.uk/spm/ext/#INRIAlign

^3^http://www.nitrc.org/projects/artifact_detect
